# Validity of the short‐form five‐item Problem Area in Diabetes questionnaire as a depression screening tool in type 2 diabetes mellitus patients

**DOI:** 10.1111/jdi.14051

**Published:** 2023-07-06

**Authors:** Donovan Tay, Marvin Chua, Joan Khoo

**Affiliations:** ^1^ Department of Endocrinology Sengkang General Hospital Singapore; ^2^ Department of Endocrinology Changi General Hospital Singapore

**Keywords:** BDI‐II, depression, depressive symptoms, distress, PAID, PHQ9, psycometric, Type 2 Diabetes mellitus, T2DM

## Abstract

**Aims/Introduction:**

Depression is prevalent in diabetes patients and associated with poor outcomes, but is currently underdiagnosed, with no firm consensus on screening methods. We evaluated the validity of the short‐form five‐item Problem Areas in Diabetes (PAID‐5) questionnaire as a screening tool for depression, comparing it with the Beck Depression Inventory‐II (BDI‐II) and nine‐item Patient Health Questionnaire (PHQ‐9).

**Materials and Methods:**

A total of 208 English‐speaking adults with type 2 diabetes, recruited from outpatient clinics, completed the BDI‐II, PHQ‐9 and PAID‐5 questionnaires in English. Cronbach's α was used for internal reliability. Convergent validity was examined with BDI‐II and PHQ‐9. Receiver operating characteristics analyses were used to identify optimal PAID‐5 cut‐offs for the diagnosis of depression.

**Results:**

All three screening tools were highly reliable, with BDI‐II, PHQ‐9 and PAID‐5 having a Cronbach's α of 0.910, 0.870 and 0.940, respectively. There was a good correlation between BDI‐II and PHQ‐9, with a correlation co‐efficient (*r*) of 0.73; and a moderate correlation between PAID‐5 and PHQ‐9, and PAID‐5 and BDI‐II, with *r* of 0.55 and 0.55 respectively (*P* values <0.01). An optimal PAID‐5 cut‐off ≥9 corresponded to both a BDI‐II cut‐off >14 (sensitivity 72%, specificity 784%, area under the curve 0.809) and a PHQ‐9 cut‐off >10 (sensitivity 84%, specificity 74%, area under the curve 0.806). Using a PAID‐5 cut‐off ≥9, the prevalence of depressive symptoms was 36.1%.

**Conclusions:**

Depressive symptoms are prevalent in people with type 2 diabetes, with the degree of distress significantly related to the severity of depressive symptoms. PAID‐5 is a valid and reliable screening tool, and a score ≥9 could prompt further confirmation for depression.

## Graphical abstract

Depression is prevalent, but neglected, comorbidity that is associated with poorer outcomes in people with diabetes. Although guidelines consistently emphasize the screening of depression, there is limited guidance on how depression should be evaluated. Part of the issue is that screenings for these psychological problems involve the use of complex questionnaires imposing both a burden on the clinician as well as the patient. Most of the studies in this area are carried out in homogenous populations. This is the first study that validates the use of the five‐item Problem Areas in Diabetes questionnaire in a multi‐ethnic population, and shows that of the five‐item Problem Areas in Diabetes questionnaire is a valid and reliable screen for both clinical depression and diabetes‐related distress in people with type 2 diabetes mellitus.

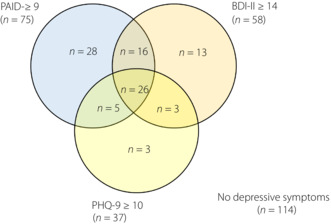

## INTRODUCTION

As many as one‐quarter of the people living with diabetes suffer from depression^1^. Depression is associated with micro‐ and macrovascular complications, cognitive impairment, decreased quality of life, increased healthcare utilization, decreased productivity, and mortality^2^, ^3^, ^4^, ^5^, ^6^, ^7^. Diabetes guidelines recommends annual screening of depressive symptoms for all people with diabetes^8^, ^9^. Validated and standardized diagnostic tools are recommended for routine clinical care to identify those who need psychosocial intervention^10^. These include the two most commonly used tools – the Beck Depression Inventory‐II (BDI‐II) and the nine‐item Patient Health Questionnaire (PHQ‐9)^11^ – as well as the 20‐item Problem Areas in Diabetes Scale (PAID‐20)^12^, which has been shown to correlate well with depressive symptoms^13^, ^14^, and can be used to measure diabetes‐related distress.

Recently, it has been shown that depressive symptoms tools are underutilized in routine clinical diabetes care^15^, and there has been no consensus on the specific assessment method to be integrated into clinical protocols. The choice of screening tool depends on the psychometric variables measured, complexity, dependence on clinician's input or patient's self‐reported symptoms and costs of licensing. A lengthy screening tool would increase the time required for administration, and this would be a burden for routine implementation in a busy clinical practice.

There are advantages of using the short‐form five‐item Problem Areas in Diabetes (PAID‐5) as an adjunct for screening of depression in diabetes, over longer and more complicated questionnaires, such as the PHQ‐9 or BDI‐II. PAID‐5 is a brief and easily administered tool in both the clinical and research setting. PAID‐5 can concurrently identify people with distress, as well as depressive symptoms.

Although the concepts of distress and depression are not interchangeable, distress is a good surrogate for depression, and can potentially mediate the association between depression and diabetes self‐management^16^. Identification of distress might facilitate early intervention and improves outcomes^17^.

We hypothesize that PAID‐5, a simple, concise version of the PAID‐20, can be used with high reliability and validity to screen for depression in people with diabetes. The present study, therefore, sought to explore the utility of the PAID‐5 questionnaire as a screening tool for depression, comparing it with two of the most commonly used and validated tools: BDI‐II and PHQ‐9. Our second aim was to estimate the prevalence of depression symptoms in people with type 2 diabetes in our population using established thresholds.

## MATERIALS AND METHODS

### Study design

Approval was obtained from our Institutional review board before carrying out the present cross‐sectional study. People with type 2 diabetes undergoing routine visits at the outpatient diabetes clinics in a regional health system were recruited. Eligibility criteria included adults (aged ≥21 years) with type 2 diabetes with whom the clinical interview was carried out in English.

Participants with a prior history of psychiatric illness were excluded, so as not to confound the results of this study with the effect of people with pre‐existing depression and/or other mental disorders. Of the eligible participants, the refusal rate was 5%. Informed consent was obtained according to institutional review board requirements. The BDI‐II, PHQ‐9 and PAID‐5, described in subsequent sections, were self‐administered to 208 English‐speaking adults. Other information was obtained through a face‐to‐face interview with the study investigators. These included patient demographics, such as age, sex, ethnicity, employment status, education level and diabetes‐related variables, such as duration of diabetes, presence of diabetes complications and type of diabetes therapy.

Biochemical data were obtained during the same visit. Serum low‐density lipoprotein and creatinine were assayed on the standard autoanalyzer Beckman Coulter UniCel® DxC 800 immunoassay system (Beckman Coulter, Inc., Brea, CA, USA) using colorimetric and enzymatic methods. Glycated hemoglobin was measured using immunoturbidimetric assay on the COBAS Integra 800 (Roche, Basel, Switzerland) with an intra‐assay coefficient variant of <1.7%, and was standardized to the National Glycated Hemoglobin Standardization Program.

Blood pressure was obtained during a brief physical examination that also included anthropometric measurements, such as height, weight and waist circumference, that were obtained by standard procedures. Body mass index was calculated in kilograms divided by the square of height in meters.

### BDI‐II questionnaire

The BDI‐II is currently one of the most commonly cited screening tools for depression in people with diabetes and has excellent psychometric qualities. The BDI‐II contains 21 items, which are based on the 4th edition of the *Diagnostic and Statistical Manual of Mental Disorders* criteria for major depressive disorder, to assess the presence and severity of depressive symptoms over the past 2 weeks. The self‐administered questionnaire assesses cognitive and somatic symptoms of depression, with each item assigned a score between 0 (“not at all”) and 3 (“most of the time”). This is added up to derive a total score between 0 and 63, with higher scores implying greater severity of depression. A score of 14–19 indicates mild depression, that of 20–28 indicates moderate depression and ≥29 indicates severe depression^18^. We used a cut‐off of ≥14 to identify depression, with a sensitivity of 82% and specificity of 89% in people with diabetes, as validated in previous studies^19^.

### PHQ‐9 questionnaire

Depressive symptoms were scored with the self‐administered PHQ‐9. PHQ‐9 is an instrument comprised of nine questions, evaluating the presence and frequency of each of the nine symptoms of the 5th edition of the *Diagnostic and Statistical Manual of Mental Disorders* criteria for depression. Each question is marked on a scale of 0–3 with 0 being “none” and 3 being “nearly every day,” which results in a total score ranging from 0 to 27. A cut‐off of ≥10 has shown a sensitivity of 88% and specificity of 88% for major depression^20^. In the present study, clinically relevant depression is defined as a total PHQ 9 score of ≥10.

### PAID‐5 questionnaire

The PAID‐5 questionnaire is an abridged scale that consists of five items instead of the full 20‐item questionnaire that constitutes PAID. It is used to measure diabetes‐related emotional distress. The five questions include: (i) feeling scared when you think about living with diabetes, (ii) feeling depressed when you think about living with diabetes, (iii) worrying about the future and the possibility of serious complications, (iv) feeling that diabetes is taking up too much of your mental and physical energy every day, and (v) coping with complications of diabetes. Each question is marked on a 5‐point scale (0 = lowest, 4 = highest), and the sum of points produces a total score that ranges from 0 to 20. A higher score indicates greater emotional distress, with high distress defined as a score of ≥8^21^. The PAID‐5 questionnaire is reliable, as defined by Cronbach's α of 0.83, sensitivity of 94% and specificity of 89% for recognition of diabetes‐related emotional distress^21^.

### Statistical analysis

Statistical analysis was carried out using R 3.2.2 (The R Foundation For Statistical Computing, Vienna, Austria). Descriptive statistics was carried out for all variables. Continuous variables are presented as the mean ± standard deviation; and categorical variables as counts and percentages. Cronbach's α was calculated to test internal reliability. Convergent validity was evaluated using Pearson's correlation to test the relationship between distress (PAID‐5) and depressive symptoms (BDI‐II and PHQ‐9). The strength of association among the two screening tools for depressive symptoms (BDI‐II and PHQ‐9) was assessed using Pearson's correlation coefficient (*r*): 0.40–0.59 indicates a moderate positive correlation, whereas 0.60–0.79 indicates a strong positive correlation and ≥0.8 indicates an excellent correlation. Receiver operating characteristic curves were used to identify the optimal PAID‐5 cut‐off in diagnosing significant depressive symptoms based on both BDI‐II and PHQ‐9 definitions. For the area under the receiver operating characteristic curves, 0.90–1.0 indicates an excellent test, whereas 0.80–0.90 indicates a good test. A *P*‐value <0.05 was considered as significant.

## RESULTS

Table 1 shows the baseline characteristics of the study population. The mean age of the study population was 57 ± 16 years. A total of 40.4% were women and 59.6% were men. The majority of the participants were Chinese (50.5%). Malays constituted 29.8%, Indians 14.4%, and 5.2% were Eurasian and other races. This distribution approximates Singaporean population demographics^22^.

**Table 1:** Sociodemographic characteristics of the study cohort at baseline

	Missing data, *n* (%)	Reference ranges	Total (*n* = 208)
Age (years)			57.1 ± 15.6
Sex			
Male (%)			59.6
Female (%)			40.4
Race			
Chinese (%)			50.5
Malay (%)			29.8
Indian (%)			14.4
Eurasian (%)			1.4
Others (%)			3.8
Smoking			
Non‐smoker (%)	3, 1.4%		77.9
Ex‐smoker (%)			10.1
Current smoker (%)			10.6
Employment status			
Employed (%)	6, 2.9%		62.0
Unemployed (%)			35.1
Education status			
Tertiary (%)	7, 3.4%		34.1
Non‐tertiary (%)			62.5
Duration of diabetes			
<5 years (%)	11, 4.8%		37.0
≥5 to <10 years (%)			17.8
≥10 to <20 years (%)			27.9
≥20 years (%)			12.5
Diabetic complications			
No microvascular complications (%)			87 (41.8)
Presence of microvascular complications (%)			121 (58.2)
No macrovascular complications (%)			145 (69.7)
Presence of macrovascular complications (%)			63 (30.3)
Diabetic treatment			9 (4.3)
Diet control			17 (8.2)
Oral glucose lowering agents			126 (60.6)
Insulin			50 (24)
Both oral agents and insulin			9 (4.3)
GLP‐1			17 (8.2)
LDL (mmol/L)		0.0–4.8	2.7 ± 1.6
HbA1c (mmol/mol)		25–46	67 ± 19
HbA1c (%)		4.4–6.4	8.2 ± 1.8
Cr (μmol/L)		65–125	100 ± 89
Weight (kg)			83.2 ± 25.7
BMI (kg/m^2^)			30.0 ± 9.0
Waist circumference (cm)			100.1 ± 20.0
Systolic blood pressure (mmHg)			141.5 ± 21.4
Diastolic blood pressure (mmHg)			76 ± 11
BDI scores			10.2 ± 9.3
PHQ‐9 scores			4.9 ± 5.2
PAID‐5 scores			6.9 ± 5.7

Continuous variables are presented as the mean ± standard deviation, whereas categorical variables are presented as percentages (%). BDI, Beck Depression Inventory; BMI, body mass index; Cr, creatinine; GLP‐1, glucagon‐like peptide‐1; HbA1c, glycated hemoglobin; LDL, low‐density lipoprotein; PAID‐5, five‐item Problem Areas in Diabetes; PHQ‐9, nine‐item Patient Health Questionnaire.

### BDI‐II

The mean BDI‐II score for the study population was 10.2 ± 9. BDI‐II was also highly reliable, with a Cronbach's α value of 0.910. The prevalence of significant depressive symptoms based on BDI‐II criterion of ≥14 was 24.5% in the study population.

### PHQ‐9

The mean PHQ‐9 score was 4.9 ± 5.2. The PHQ‐9 was highly reliable, with a Cronbach's α value of 0.870. The prevalence of significant depressive symptoms based on the PHQ‐9 criterion of ≥10 was 13.9% in the study population.

**Figure 1:**
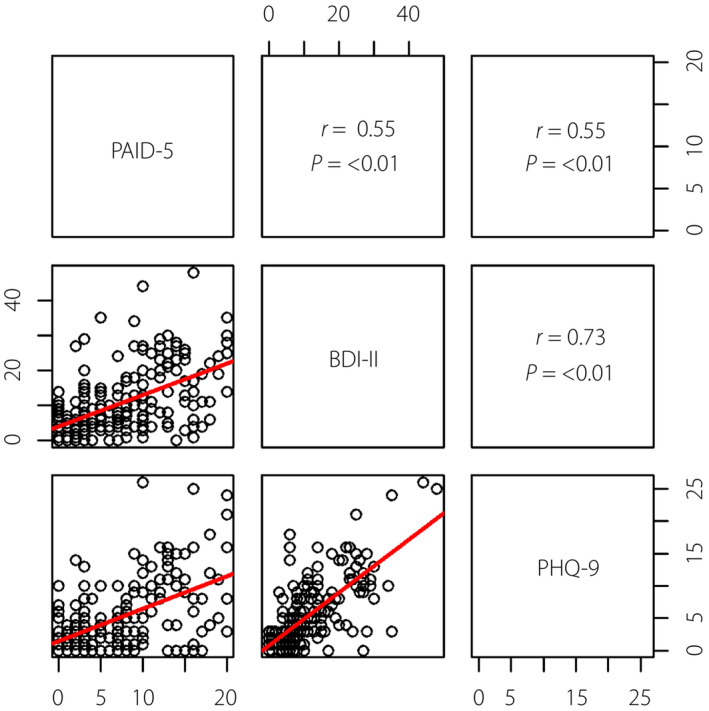
Relationship between the Beck Depression Inventory‐II (BDI‐II) score and nine‐item Patient Health Questionnaire (PHQ‐9) score in the study population showing a strong correlation between the BDI‐II and the PHQ‐9 (*r* = 0.73; *P* < 0.01). The relationship between the BDI‐II score and the five‐item Problem Areas in Diabetes (PAID‐5) score in the study population showing a moderate correlation between the PAID‐5 and the BDI‐II (*r* = 0.55; *P* < 0.01). The relationship between the PHQ‐9 score and the PAID‐5 score in the study population showing a moderate correlation between the PAID‐5 and the PHQ‐9 (*r* = 0.55; *P* < 0.01).

There was a good correlation between BDI‐II and PHQ‐9, with a Pearson correlation coefficient of 0.730 (*P* < 0.01). There was a moderate correlation between PAID‐5 and BDI‐II, as well as between PAID‐5 and PHQ‐9 (both *r* = 0.55, *P* < 0.01; Figure 1).

### PAID‐5

The mean PAID‐5 score for the study population was 6.9 ± 5.7. PAID‐5 showed a Cronbach's α value of 0.940, indicating that it was highly reliable. The prevalence of distress was 41.3% (using conventionally established PAID‐5 criterion of ≥8 for distress). A total of 36.1% of the study population were identified using the PAID‐5 criterion of ≥9 (the optimal cut‐off used to determine significant depressive symptoms based on BDI‐II and PHQ‐9 definitions).

**Figure 2:**
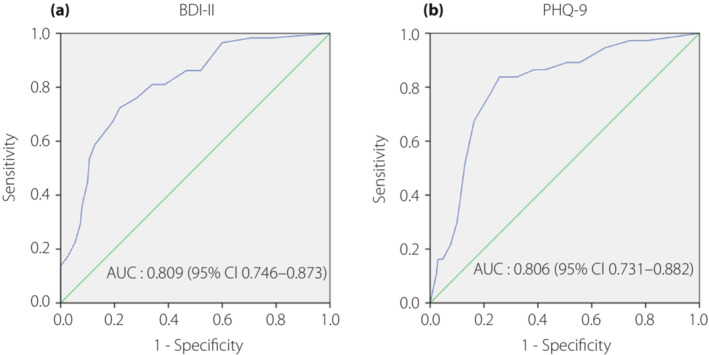
Receiver operating characteristic curves show the diagnostic performance of the five‐item Problem Areas in Diabetes in discriminating between respondents with significant depressive symptoms as defined by (a) Beck Depression Inventory‐II (BDI‐II) cut‐off of ≥14 and (b) nine‐item Patient Health Questionnaire (PHQ‐9) cut‐off of ≥10 from those without. Receiver operating characteristic curve plots the true positive rate (sensitivity) against the false positive rate (1‐specificity) for different cut‐offs. The curved line represents the area under the receiver operating characteristic (AUC) for the five‐item Problem Areas in Diabetes, whereas the straight diagonal line represents chance (AUC of 0.5). CI, confidence interval.

The ability of the PAID‐5 questionnaire to determine the presence of significant depressive symptoms based on BDI‐II and PHQ‐9 conventional thresholds was assessed by receiver operating characteristic analyses. With PAID‐5, the area under the curve for identifying significant depressive symptoms by BDI‐II definition was 0.809 (95% confidence interval 0.746–0.873), whereas the area under the curve by PHQ‐9 definition was 0.806 (95% confidence interval 0.731–0.882). A threshold of 9 for the PAID‐5 score results in an optimal combination of sensitivity 72% and specificity 78%, positive predictive value of 56%, negative predictive value of 88%, and accuracy of 76% when compared against the BDI‐II. Similarly, the optimal cut‐off point for the PAID‐5 was also 9 when compared against the PHQ‐9, with a sensitivity of 84% and specificity of 74%, positive predictive value of 41%, negative predictive value of 95%, and accuracy of 76% (Figure 2).

**Figure 3:**
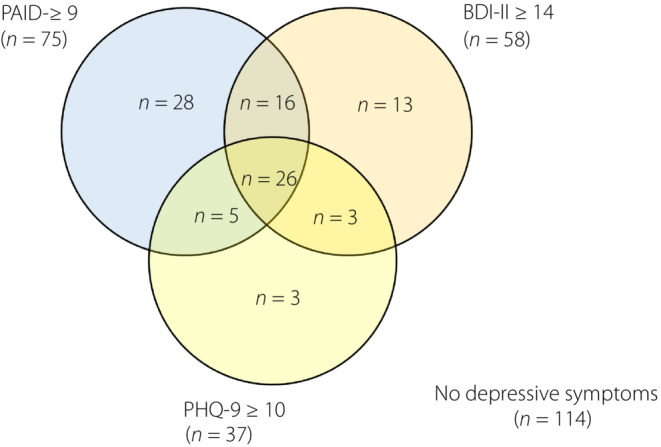
The prevalence of individuals with depression according to diagnosis by five‐item Problem Areas in Diabetes (PAID‐5) criterion ≥9, Beck Depression Inventory‐II (BDI‐II) criterion ≥14 and nine‐item Patient Health Questionnaire (PHQ‐9) criterion ≥10.

Using the PAID‐5 ≥9 criterion, six (16%) individuals were missed when compared against the PHQ‐9 definition (cut‐off ≥10), and 16 (28%) individuals were missed when compared against the BDI‐II definition (cut‐off ≥14). Of the individuals identified by PAID‐5 (using cut‐off ≥9), 84% and 72% were also concordantly identified using the PHQ‐9 and BDI‐II, respectively. Of note, only 26 out of 94 individuals (28%) were consistently classified based on the three definitions using PAID‐5, BDI‐II and PHQ‐9 scores (Figure 3).

## DISCUSSION

We showed that PAID‐5 is a reliable screening tool for depression in people with diabetes. Convergent validity was also shown by a significant correlation with the BDI‐II and PHQ‐9, both widely used and well‐validated^11^ measures of depressive symptoms. Translated versions of the full 20‐item PAID have been validated in different homogenous societies^23^, ^24^, ^25^, ^26^, ^27^, ^28^, ^29^, ^30^. Both the English and the Chinese version of 20‐item PAID had been validated in Singapore, but not the short version used in the present study^31^, ^32^. This is the first study to confirm its utility in a multi‐ethnic Singaporean population. Psychometric evaluation of the Korean version of the PAID‐5 in a Korean population showed that the short version was as reliable as the PAID‐20, with the Cronbach's α of the PAID‐20 when analyzed as a one‐factor structure shown to be 0.94, whereas that of PAID‐5 was 0.87, and that the reliability of the PAID‐20 decreases when analyzed as a two‐to‐four factor structure^30^.

The present study showed that depressive symptoms are prevalent in a multi‐ethnic population screened during routine follow up for type 2 diabetes mellitus. In the Diabetes Attitudes, Wishes and Needs second study (DAWN‐2), a large multinational study involving 17 different countries, the prevalence of depression was 25.5%^1^, similar to the prevalence in the present population. In a meta‐analysis to determine the prevalence of clinically relevant depression in adults with type 1 or 2 diabetes, the lifetime prevalence of major depression was 28.5%, with a mix studies with self‐reported depression symptoms scales and diagnostic interviews^33^.

Despite the high prevalence, depression is frequently underrecognized and undertreated by healthcare workers and caregivers^34^. Lustman *et al*.^34^ showed that clinical depression was recognized in only one‐third of depressed adults with diabetes, and only half received treatment for their depression. This might be contributed by a lack of awareness of available screening tools, or the difficulties of these tools being too long and unwieldy to administer during clinic consults. As psychological comorbidities can interfere with the ability of patients to self‐manage their disease, appropriate and timely intervention facilitates self‐care, reduce complications, and improves quality of life and life expectancy of people with diabetes^33^. Even though calls for improved detection of these neglected comorbidities have been emphasized in international guidelines on diabetes, limited guidance is available for the method of detection.

The present study adds to the ongoing study of the properties of various screening instruments and their validities in diabetes^35^. We found that the PAID‐5 only correlated moderately with the depressive symptoms scores (BDI‐II and PHQ‐9). This is likely related to its brevity and different psychometric construct It has previously been shown that the correlation between the full version PAID‐20 and BDI was moderate (*r* = 0.579), and similarly between the PAID‐20 and PHQ‐9 (*r* = 0.50)^13^, ^14^. When compared with the BDI‐II, the PAID‐5 was falsely negative in 28% and falsely positive in 16%. When compared with the PHQ‐9, the PAID‐5 was falsely negative in 16% and falsely positive in 26%. A prior study comparing PAID‐20 with the Centre for Epidemiologic Studies Depression Scale, a depressive symptoms score, showed PAID‐20 (threshold of 40) misclassified 19.8% (*n* = 124) of participants with diabetes and depressive symptoms as normal, and misclassified 2.4% (*n* = 15) of participants as having depression^16^. Of note, this cut‐off of 40 on the PAID‐20 is arbitrarily defined as one standard deviation from the mean of the European study population with diabetes and not defined based on clinical outcomes, such as depression^36^. When bench‐marked against instruments that measure depressive symptoms, a higher cut‐off of ≥9 (rather than the conventional cut‐off of 8) provided an optimal balance between sensitivity and specificity in our population. Using this threshold, the positive predictive values were low, 56% (BDI‐II criteria) and 41% (PHQ‐9 criteria), respectively, which might limit its utility as a diagnostic tool. Conversely, the high negative predictive value of the PAID‐5 of >88–95% supports its use as a screening tool for depressive symptoms.

Although BDI‐II and the PHQ‐9 have been compared in adults with other chronic diseases, such as rheumatoid arthritis^37^ and obesity^38^, the comparison of the psychometric properties between the two in diabetes is limited. The present study showed that although both BDI‐II and PHQ‐9 had a good correlation, only 50% of those identified with significant depressive symptoms using BDI‐II established thresholds were identified using PHQ‐9 definitions, showing that these two measures were dissimilar.

There were several limitations to the present study. We did not compare the PAID‐5 with the gold standard measurement of depression, namely, the standardized diagnostic interview for depression^39^. Therefore, these questionnaires identified depressive symptoms rather than depression. Using depressive symptom scores might overestimate the diagnosis of depression. However, most interventional studies of diabetes and depression used depressive symptom scores to measure improvement in outcomes, rather than a diagnostic interview^40^, ^41^. Similarly, epidemiological studies showed that the association of poor diabetes outcomes, such as microvascular and macrovascular complications, and mortality, were with depressive symptom scores rather than a diagnosis of depression made by a structured interview^4^, ^5^. The presence of symptoms below the thresholds for clinical depression is also associated with poorer outcomes^42^. The evidence strongly supports the clinical utility and intervention based on objective evaluation of depressive symptoms, compared with a dichotomous diagnosis by a structured interview.

The present study population was relatively small and, as only English‐speaking individuals were enrolled, this might have introduced selection bias. However, the present findings are consistent with prior larger studies^1^. Furthermore, our refusal rate was low, at only 5%.

The present population was derived from a regional center in which the prevalence of depressive symptoms might not be representative of the community. In particular, our population included people with long‐standing, poorly controlled diabetes with a high rate of complications, which might lead to higher levels of depressive symptoms. However, the prevalence of distress and depressive symptoms was similar to that in the DAWN‐2 study^1^. Also, a study in primary care clinics in Malaysia (which has a multi‐ethnic population, but higher predominance of Malays) showed a similar prevalence of distress of 49.2% (using the Diabetes Distress Scale), but a higher prevalence of depressive symptoms of 41.7% (using the PHQ‐9)^43^. The present study was a cross‐sectional study, and we therefore cannot infer causal or temporal relationships between distress and depression. The false negative rate of 16% (compared with the PHQ‐9 criterion) or 28% (compared with the BDI‐II criterion) is a potential limitation for use of PAID‐5 as a screening tool.

Depressive symptoms are prevalent in people with type 2 diabetes mellitus. The degree of distress is significantly related to the severity of depressive symptoms. PAID‐5 is a valid and reliable screening tool for depressive symptoms, although its utility might be limited by a low positive predictive value. We suggest further evaluation and management of depression and distress in people with a score ≥9.

## FUNDING

The study was supported by the Changi General Hospital Grant. The funder was not involved in study design, data collection, data analysis, manuscript preparation and/or publication decisions.

## DISCLOSURE

The authors declare no conflict of interest.

Approval of the research protocol: The study was approved by SingHealth's Centralized Institutional Review Board.

Informed consent: Written informed consent was obtained from the participants of the study.

Registry and the registration no of the study/trial: N/A.

Animal studies: N/A.
